# Remarks on the Yellow Fever

**Published:** 1841-06-05

**Authors:** A. W. Upshur


### ORIGINAL COMMUNICATION.

### REMARKS ON THE YELLOW FEVER.

BY A. W. UPSHUR, M. D.

(Concluded from page 279.)

To the Editors of the Medical Examiner.

The memoir of Dr. Potter, to which we have previously alluded, affords abundant evidence that yellow fever often appears in situations in which there is no possibility of its having been been produced by the presence of the sick.

Mr. Ellicott, in his journal, (of a voyage down the Ohio in November, 1796,) is so explicit and intelligent on this subject, that no reasoning can shake his statement. His words are these: "I arrived at Gallipolis at eleven o'clock in the morning. This village is a few miles below the great Kenhaway, on the west side of the Ohio river, situated on a high bank; it is inhabited by a number of miserable French families, many of which, this season, fell victims to the yellow fever. The mortal cases were generally attended with black vomit. This disease certainly originated in the town, and, in all probability, from the filthiness of inhabitants, added to an unusual quantity of animal and vegetable putrefaction in a number of small ponds and marshes within the village. The fever could not have been taken from the Atlantic States, as my boat was the first that descended the river after the fall of the waters in the spring; neither could it have been carried from New Orleans, as there is no communication, at that season of the year, from the latter to the former place; moreover, the distance is so great, that a boat would not have time to ascend the river, after the disorder appeared that year in New Orleans, before the winter would set in."

Dr. Potter received a more particular account of the same fever from Major Prior of the army, who witnessed the rise and progress of the disease which is given above. Some particulars, not there mentioned, are stated here. The sick generally died with the black vomit; they were often yellow before death, and almost always afterwards. As some decisive measures became necessary to save the rest of the troops, a ditch was cut; what little water remained was conveyed off, and the whole surface covered with fresh earth. Not a man was seized with the worst form of the disease after the work was finished; and the sick were not a little benefitted, for they generally recovered, (though slowly,) because the fever became a common remittent, or gradually assumed the intermitting form. Frost put a stop to the fever in every form.

The following is equally explicit as to the indigenous origin of yellow fever. At a village called New Design, fifteen miles from the Mississippi, and twenty from St. Louis, containing about forty houses, and two hundred inhabitants, in the summer and autumn of 1797, the yellow fever destroyed fifty-seven of the inhabitants, or more than one-fourth. No person had arrived at this village from any part of the country where the disease had prevailed, for more than twelve months preceding its appearance. Dr. Watkins resided in the village at the time, and, having seen the disease in Philadelphia, testifies to the facts. Dr. Potter also mentions some cases of yellow fever which occurred in a house near Baltimore, in September, at a time when that city and the adjacent country were remarkably healthy. Struck with the circumstance he examined the premises narrowly, and discovered *that the cellar contained water, which had remained there from the first week in June, the country having been then inundated with rain. The cellar being useless, was closed, and the only vent for the gas arising from it was through the floor, which was open in several places. At his solicitation, the survivors were removed, and convalesced from that time.* The tragedy, however, did not terminate here. The owners of the house, anxious to retrieve its character, hired two men to empty the cellar. This labour was performed in one day, by ripping up the floor and drawing off the water by means of a pump. In a few days after they both sickened and died with the usual symptoms of yellow fever. The same writer says, during the embargo in 1808, when no foreign sail whitened our waters, in August the yellow fever commenced its ravages at Fell's Point.

The commission named by the Academy of Sciences in Paris, to decree the prizes on medicine and surgery, established by M. Montyon, awarded that of ten thousand francs to Dr. Chervin for his researches on yellow fever. The following extract is from their report. In the year 1814, Dr. Chervin began his researches at Guadaloupe, and, not content with visiting every patient with yellow fever that he had access to, he made autopsical examinations on more than five hundred bodies in fifteen months, at Point-a-Petre; but this was not enough to satisfy him. He continued his researches in America for eight years, visiting every place in which the yellow fever appeared, traversing, in this time, not less than thirty thousand leagues. On his return to Europe, the fever was raging in Spain, and he immediately repaired to that country, and visited every place infected with the disease: the result of all his observations go to prove that yellow fever is not contagious; and this opinion is strengthened by the opinions of five hundred out of six hundred and thirty American and Spanish physicians, whose answers to his questions he is now in possession of.

The following experiments are strong evidences in favour of the non-contagious character of this disease. M. Guyon took the shirt of a patient, who had just died of the fever, and put it on *whilst it was still soaked with sweat; at the same time he was inoculated with the yellowish matter from blisters*, which the patient had had shortly before his death. *He wore the shirt for twenty-four hours*, during which time he was constantly observed by medical witnesses. Two days afterwards *he drank about two ounces of black fluid vomited by a patient* who died on the following day. *Another portion of the same fluid was rubbed into his arms, and he was also inoculated with it. Immediately after the death of the second patient, he put on his shirt also, which was much stained with black fluid, and lay in his bed, where he remained six hours and a half. He then opened the body of the first patient, whose stomach was found filled with black fluid, inflamed, and with the mucous membrane ulcerated; he was again inoculated with the black matter, and pieces of the stomach were applied over the wounds*, which, after twenty-four hours, were found inflamed and very painful. *After three days, these symptoms having disappeared, M. Guyon was perfectly well.* All these experiments were made in presence of medical officers of the station, and Lieut. General Daugelot, governor of Martinique, vouches for their authenticity.

This disease has even been produced at sea, in circumstances in which there was not the slightest ground to doubt its generation on board the ship. The United States ship of war, General Green, sailed from Newport, in Rhode Island, on the third of June, 1799. At sea the yellow fever broke out. The fever did not exist at Newport previous to her departure. That town is too far north for the appearance of the yellow fever, except in very hot summers, or so soon in the year, in the hottest. The ship touched no where, and had no communication with any vessel, until she arrived at Havana, after the appearance of the disease. The Burbridge, Indiaman, sailed from England for Madras and Bengal on the fifteenth of April, 1792. On the 26th of May she crossed the line twensix degrees west longitude. The mercury ranged from eighty to eighty-six. The weather was extremely sultry, with frequent rains. In this state of things the yellow fever broke out, although she had touched at no port, nor had communication with any vessel.

The following cases were related to me by an officer of the United States Navy, who witnessed them on board a ship to which he was then attached. In the year 1822, an American sloop of war sailed from the United States on a cruise to the West Indies and Gulf of Mexico; she passed the winter at different ports near the head of the Gulf of Mexico. In the following spring the ship was ordered to Tampico to receive on board a number of Americans and others, who had previously been residing in the city of Mexico as state prisoners. On their release from prison, where they had been half-clothed and badly fed, they marched from the city of Mexico to Tampico, principally on foot, over a broken and rugged country, and under a hot tropical sun; they were consequently much exhausted and emaciated on their arrival at Tampico. They remained about a week in that city, before they embarked, living freely and indulging to excess in the fruits and vegetables of that country. The day following their embarkation, one of them, who was a strong hearty young man, was attacked with severe pain in the head and back; he was questioned by the surgeon of the ship as to what he had been eating, and replied that he had eaten a great deal of green sugar cane, which he thought was the probable cause of his disease. The surgeons pronounced his case to be yellow fever, and treated it accordingly. About eight o'clock on the second night of his attack, he became delirious, calling constantly for cold water, and complaining, in a strain of delirium, that although rivulets were running at his feet, no one would give him a drop. He had been previously removed from the birth-deck, into a cool and airy place under the top-gallant forecastle. At ten o'clock his cries became so loud and distressing as to attract the attention of the narrator, who was at that time in charge of the deck, and who immediately went forward and gave him about a pint of cold water, the whole of which he drank at a draught. He had scarcely swallowed it before it returned, followed by a quantity of black matter resembling coffee grounds. From that time he continued to drink cold water and to vomit, for the space of four hours, at which time he died a perfect madman, having suffered the most excruciating pain. *He had remained on board the ship for three days, had the disease in its most malignant form, and not an individual took it from him.* The ship sailed from Tampico to Havana, in the island of Cuba. On her passage, of twenty-one days, the crew suffered extremely for want of provisions and water, it being impossible to procure either in Tampico, in consequence of the revolution which was then raging, and the consequent demands for the supply of the army. On her arrival in Havana, about the first of August, with an emaciated and weather beaten crew, it was found that the yellow fever was raging in the city and in the harbour to a frightful extent, thirty-five or forty dying daily on shore, and about an equal proportion of those who were afloat. The sloop of war anchored within two cables length of a Spanish eighty gun ship, on board which the fever had been raging for many weeks. *Official visits were exchanged by the two ships, and the officers of each occasionally visited the other, during the time that the Spanish ship was daily losing from three to five of her crew, and no case of fever occurred on board the sloop of war which could in any way be ascribed to her intercourse with the other ship.*

But a few days previous to the departure of the sloop of war from port, her launch, with eighteen men and two officers, was sent, at sun-rise, some miles up the river for fresh water, and, owing to an accidental detention, did not reach the ship until about 2 o'clock, P.M. The day was excessively hot, and the officers and crew of the launch had fasted all day. On her arrival alongside, at 2 o'clock, P.M., the senior officer came over the gangway, holding one hand on his head and the other to his back, and appeared to be perfectly stupid, saying to the narrator, who met him at the gangway, "report my boat and take me below; my head is bursting, and my back is broken." He was accordingly carried below, laid upon a locker, and immediately became insensible. The surgeon of the ship was at that moment engaged; as soon as practicable, however, he was copiously bled, and forced to take thirty grains of calomel. In half an hour he went to sleep, and slept profoundly until eight o'clock next morning, when two copious and very dark evacuations were produced in quick succession, after which the patient said he felt perfectly well, with the exception of debility; and the next day he was on duty.

Three days thereafter, the narrator was himself attacked precisely in the same way, and from the same cause. The same remedies were promptly administered, and relief speedily obtained. He was also on duty on the third day after his attack. In about ten days thereafter the ship sailed for the United States, and these were the only cases of yellow fever that occurred on board of her during a cruise of nearly a year in the West Indies and Gulf of Mexico; and circumstances plainly show that these did not originate in contagion, else their attendants, and others in the ship, would not have escaped.

It is perhaps needless to add more to the abundant evidence which has already been cited. Suffice it to say, if any dependence is to be placed in the description of yellow fever by the medical gentlemen of cities—if any is to be placed on the decisions of the physicians of the country, founded on a comparison of cases occurring in their practice with these descriptions, and the occasional advantage of seeing for themselves on their visits to the sea-ports—fevers occasionally occur with the symptoms of yellow fever, including the black vomit, in situations in which it is impossible to believe the disease did not originate on the spot. We have, then, unquestionable evidence that the yellow fever often is, and may at any time be produced in certain circumstances, without the presence of persons previously affected. Again: When the sick are carried out of these circumstances, or removed beyond the influence of the cause generated in them, no person who approaches them will be affected.

This position is fully established by many instances, in which, when the sick are brought out of situations favourable to the spread of the disease, into a pure atmosphere, the disease is not propagated, no one in the pure air being affected by the presence of the sick. Experience has long since shown that this disease, when carried from a city into the pure air of a neighboring village, does not spread; *nay, that in the very city in which its fatal ravages are felt, there are parts in which, if a sick person be carried into them, the disease will not spread.*

We find, therefore, that yellow fever does not possess the characteristics of a contagious disease: viz., that of affecting a person but once, and that of spreading until it becomes universal, even in circumstances favourable to its extension unless stopped by insulating the sick. Moreover, great numbers placed near the sick, attending on them, escape entirely; it is produced in certain circumstances wherever they occur, without the presence of any person previously affected; and when the sick are carried out of these circumstances, no person who approaches them will be affected. We must, therefore, conclude that the yellow fever is not propagated by a volatile contagion.

In opposition to a conclusion resting on such strong grounds, the doctrine of the contagious nature of yellow fever rests solely on the appearance of the disease after a sick person has been introduced into a town, and on the rapid spreading of the disease. As to the first, it has been shown that the disease has originated in certain circumstances without the introduction of the sick. Therefore there is a cause, independent of a communication with them, or of contagion derived from them; and that this cause is a gas, produced in such circumstances, is evident from the effect of exposure to a wind blowing over the places in which those circumstances exist, as well as from the effect of cutting off the source of it by draining, filling up, inundating, &c. It is also well known that this disease very often does not follow the introduction of the sick, and, therefore, such introduction is not alone sufficient to produce it. It is further known, that it never *spreads* after the introduction of the sick into a town, unless the town be very filthy, and the temperature be very high. These are the very circumstances in which the disease has been shown to have originated; consequently the *gas* produced in these circumstances is the *cause*, and the introduction of the sick is not.

There is nothing in this circumstance to indicate the *nature* of the cause; all that we can infer is, that the cause is extensive in its operation. The gas which arises in the circumstancess in which this disease always appears, is capable of being applied to any number of persons at one moment; nay, if they be in the direction of the wind blowing over the spot in which these circumstances exist, and there be no obstruction, it cannot be avoided. It is, therefore, better applied to the rapid production of the disease than contagion; any contagion, ascertained to exist, being incapable, as is well known, of affecting a person at the distance of twenty feet. The former, indeed, is capable of the rapid production of the disease, and contagion is not. There is evidently, therefore, no foundation for the doctrine in either of these circumstances; nothing to produce a doubt of the correctness of the conclusion that the disease is not contagious.

The yellow fever appears in those places noted for the appearance of ordinary bilious fever, and, we have fully shown, is produced by the same cause, viz., *miasmata.* It is more violent than the ordinary fever, and it appears only when the causes of ordinary fever are present in great force. Before the cause has acquired that degree of force necessary to the production of yellow fever, bilious fever is common. It gradually becomes more and more violent, as the weather becomes more and more favourable to the production of the common cause. The nature of the fever becomes the subject of warm contention; some declaring it to be yellow fever, others asserting it to be only the ordinary fever, rendered more violent by the increased heat of the weather; at length all admit the yellow fever to exist.

Even after this is universally admitted, at a little distance from the chief sources of the miasmata, in the higher parts of the city, cases of the ordinary bilious fever are common; nay, it is impossible to decide, in the first hours of an attack, whether the disease is the ordinary bilious fever, or will turn out to be yellow fever; many cases of the latter ending in the former, after a violent commencement, and many pronounced ordinary bilious fever terminating with all the symptoms of malignant yellow fever. Not only do both exist at one time, under the operation of one cause, in different parts of the same city, but of those in the very middle of the most sickly part, some have only the ordinary fever. Finally, throughout the whole course of the epidemic, warm disputes often occur respecting the nature of particular cases; *plainly showing the impossibility of drawing any intelligible distinction between them.*

In the country, as in cities, where the circumstances productive of the cause of bilious fever are in great force, the fever becomes malignant, and cases often occur not to be distinguished from the yellow fever. And there is no symptom of yellow fever that does not occasionally occur in the neighbourhood of marshes, ponds, and rivers. In fine, bad cases of bilious fever may, by mismanagement, be made to assume all the symptoms of yellow fever; and yellow fever, by judicious treatment, assumes, on the road to health, the symptoms of the ordinary bilious intermittent.

We have, therefore, as good reasons for believing that yellow and bilious fevers are one, as that remittent and intermittent fevers are so.

